# The evolution and mechanism of bacterial and archaeal ESCRT-III-like systems

**DOI:** 10.1016/j.sbi.2025.103111

**Published:** 2025-07-06

**Authors:** Tom A. Williams, Harry H. Low

**Affiliations:** 1 Department of Life Sciences, University of Bath, Bath, UK; 2 Department of Infectious Disease, Imperial College, London, UK

## Abstract

The endosomal sorting complex required for transport-III (ESCRT-III) system is an ancient protein family involved in membrane remodelling. Recent phylogenetic and structural analyses reveal its conservation across the tree of life, including bacteria and archaea, suggesting an evolutionary origin predating the last universal common ancestor. These findings underscore the importance of the ESCRT-III superfamily to our origins, particularly with the recognition of their contribution to eukaryogenesis through the Asgard archaea lineage. Bacterial systems, often with a single ESCRT-III–like protein, offer a simple model for understanding how ESCRT-III can function as both membrane sensor and sculptor. This review explores the structural dynamics, evolutionary trajectories, and biological significance of ESCRT-III in bacteria and archaea. We describe how ESCRT-III polymerises and assembles conserved filaments with the coating of flat or positively curved membranes prevalent, at least *in vitro*. Finally, we highlight common mechanistic principles and unique adaptations that enable ESCRT-III systems to support diverse cellular processes across evolutionary domains.

## Introduction

**Figure 1:**
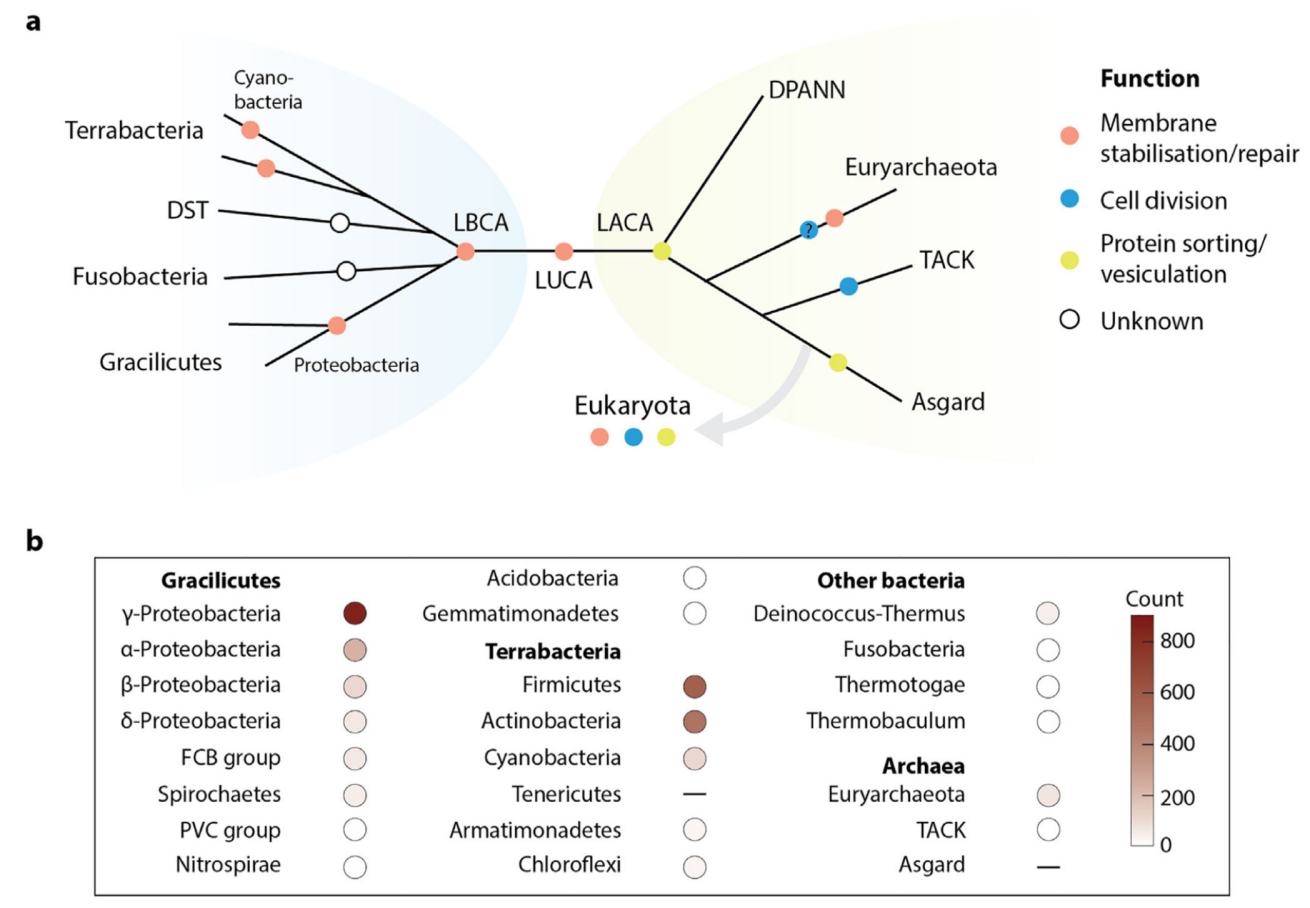
Evolution of ESCRT-III function across the tree of life. **(a)** Phylogeny of ESCRT-III family proteins across Archaea, Bacteria, and Eukaryota suggests their primordial presence in the LUCA, where they likely functioned in membrane repair. This role has been retained in many lineages, but ESCRT-III proteins have also evolved new functions during the history of life, including roles in protein sorting and vesiculation (in archaea and eukaryotes) and in cell division. ESCRT-III, endosomal sorting complex required for transport-III; LUCA, last universal ancestor; LACA, last Asgard common ancestor; LBCA, last bacterial common ancestor; DST, Deinococcus-Thermus, Synergistetes, and Thermotogae. **(b)** Heatmap showing the phyletic spread of PspA across core bacterial and archaeal lineages, adapted from Ref. [5]. The colour gradient represents the number of homologues identified within each lineage.

ESCRT-III (endosomal sorting complex required for transport-III) is an ancient and widely distributed family of polymer-forming proteins involved in membrane binding and remodelling. It was initially identified for its role in multivesicular body cargo sorting, where it typically combines with three other conserved multisubunit complexes including ESCRT-I, ESCRT-II, and ESCRT-IV (Vps4). Subsequently, it has been found contributing to diverse eukaryotic processes including cell division, membrane repair, and viral budding [1]. The broader significance of ESCRT-III became evident when shown to mediate cell division in Crenarchaeota, a group of archaea within the TACK (Thaumarchaeota, Aigarchaeota, Crenarchaeota, and Korarchaeota) superphylum [2]. More recently, phylogenetic analyses have shown ESCRT-III proteins to be conserved across the tree of life, with an ancestry extending to the last universal common ancestor (LUCA; Figure 1a) [3,4]. The ESCRT-III family includes PspA and Vipp1 (IM30) in bacteria, Cdv and ESCRT-III in archaea, and multiple ESCRT-III paralogues in eukaryotes.

In this review, we focus on prokaryotic ESCRT-III systems, which have fewer components than their eukaryotic counterparts, potentially making them simpler models to study. We explore the evolution of ESCRT-III genes, including their presence in prokaryotes, and examine their known cellular functions. Based on this, the possible role of ESCRT-III in the LUCA is discussed. Finally, a comparison of bacterial, archaeal, and eukaryotic systems highlights the conserved principles underlying ESCRT-III–mediated membrane remodelling.

### The evolution of ESCRT-III and its functional diversification

**Figure 2:**
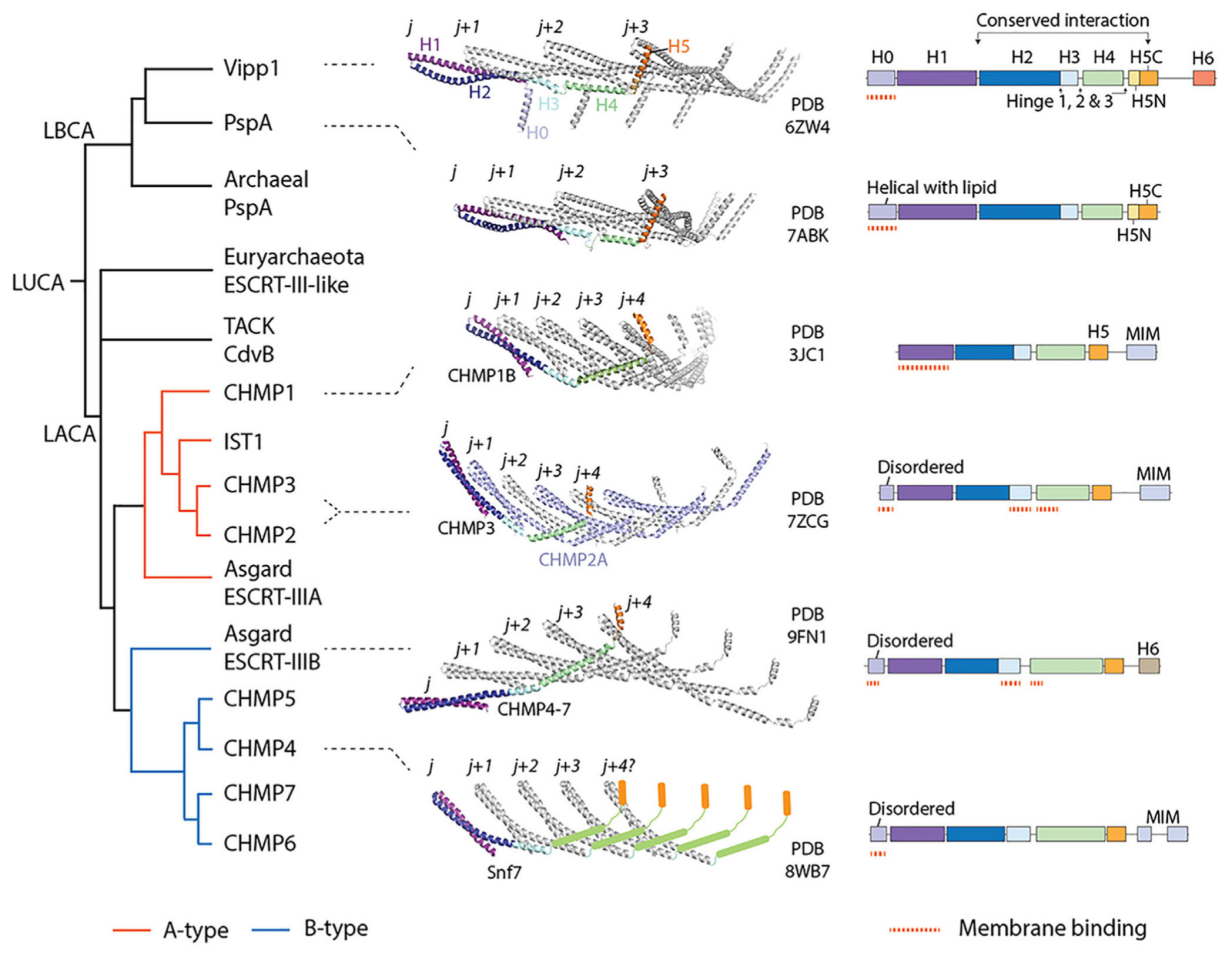
Phylogeny (left), conserved protofilament polymerisation mechanism (middle), and protein domain organisation (right) of the ESCRT-III superfamily. PspA and Vipp1 lineages have an extended H5 comprising two helical motifs, H5N and H5C. Whilst H5C interfaces with the hairpin of subunits in the same protofilament, H5N simultaneously interfaces with the hairpin of subunits from a neighbouring protofilament. Asgard ESCRT-IIIA (CHMP1-3) and ESCRT-IIIB (CHMP4-7) are A-type and B-type ESCRT-III paralogues, respectively [24,34]. For Snf7, H4 and H5 are modelled in the predicted open conformation [59]. Phylogeny is a schematic representation based upon recent analyses [3,18,83]. ESCRT-III, endosomal sorting complex required for transport-III; LUCA, last universal common ancestor; LACA, last archaeal common ancestor; LBCA, last bacterial common ancestor.

In bacteria, ESCRT-III family proteins known as PspA are widely distributed across many major lineages, with notable concentrations in Proteobacteria (Gracilicutes), Actinobacteria, Firmicutes, and Cyanobacteria (Terrabacteria) (Figure 1a and b) [5,6]. Across these lineages, PspA function appears generally conserved, typically localising to the inner membrane where it supports membrane maintenance and repair in response to cell envelope stress [7]. PspA activation triggers include biological or environmental stresses such as phage infection [8] or antibiotic exposure [7] or internal factors such as protein biogenesis and secretion [9]. PspA often exists in operons, operating in combination with accessory proteins, with the model systems in *Escherichia coli* [7], *Bacillus subtilis* [9–12], and *Mycobacterium tuberculosis* [13,14] being the most studied. Recent genomic analyses now identify multiple novel partners of PspA including the Toastrack domain, transcriptional regulators like PadR-wHTH, and Band-7 domains all with undetermined roles [5,6]. Notably, a Vps4-like ATPase that plays a conserved role in regulating ESCRT-III assembly dynamics in archaeal and eukaryotic systems appears absent in bacteria, suggesting alternative mechanisms for PspA polymerisation regulation [5]. Whilst not homologous to Vps4, in Proteobacteria, PspA is typically located in an operon with the AAA+ ATPase PspF, which has dual function–it inhibits PspA assembly when bound but functions as a transcription factor that upregulates the *psp* operon once dissociated from PspA. Where PspA is transcribed as a monocistronic unit, as occurs in over half of cyanobacteria, this may reflect its capability to function independently as both membrane stress sensor and effector [6]. Importantly, cyanobacteria and chloroplasts also incorporate the conserved PspA paralogue Vipp1–an essential protein implicated in membrane maintenance and repair, alongside thylakoid biogenesis [15,16]. PspA homologues have been acquired by some Archaea (Figure 2), apparently by early horizontal gene transfer from bacteria into the Methanotecta common ancestor, including halophilic archaea. Although the functions of these PspA genes are not well characterised, *Haloferax* PspA is upregulated by salt stress, consistent with a role in membrane response to environmental insult, like in bacteria [17].

In Archaea, ESCRT-III homologues have been found in all major lineages except the DPANN superphylum (Figure 1a) [3]. As a defining feature, these homologues occur alongside the ATPase Vps4, which drives the nucleotide-dependent disassembly of ESCRT-III. The origins of Vps4 trace to the first archaeal common ancestor, with it being well established by the last archaeal common ancestor (LACA) [3,18]. Proteins with winged helix-turn-helix motifs that are characteristic of modern ESCRT-II also date back to LACA. Therefore, the presence of Vps4 and components relating to ESCRT-II supports protein sorting and membrane vesiculation capabilities in early Archaea [19], possibly orchestrating the release of extracellular vesicles like in Crenarchaeota where vesicles transport chromosomal and plasmid DNA between *Sulfolobus* cells [20]. In TACK archaea, early ESCRT-III duplicated to become the Cdv system co-opted for cell division presumably due to the loss of the ancestral FtsZ-based division system [2,21]. This contrasts with the Asgard lineage which retained FtsZ, likely to coordinate cell division. Notably, ESCRT-III–mediated cell division appears to have evolved independently at least two other times, with different mechanisms in each case–in Thermoplasmatota (within Euryarchaeota) where some lineages lack FtsZ [19] and in eukaryotic cells [1]. In Asgard archaea, ESCRT-III underwent a key gene duplication, creating the paralogues ESCRT-IIIA and ESCRT-IIIB. These subsequently formed part of an increasingly complex Asgard ESCRT system that included relatives of ESCRT-I, -II, and -IV coupled with ubiquitin machinery [19,22–24]. However, whilst this suggested the possible presence of a sophisticated eukaryotic-like endomembrane system and sorting machinery for determining protein fate, intracellular membrane compartments were not apparent in the first glimpse of Asgard cell ultrastructure [25]. After eukaryotes diverged from Asgard archaea, each ESCRT-IIIA and ESCRT-IIIB gene family underwent multiple gene duplications to yield the distinct A-type (relating to yeast Vps2/24/46) and B-type (Vps20/32/60) ESCRT-III subfamilies observed in eukaryotes today (Figure 2) [24].

Such advances in our understanding of ESCRT-III evolution allow us to infer its function in the LUCA. ESCRT-III likely did not mediate cell division due to the absence of Vps4 and the likely presence of an early FtsZ homologue [4,26]. In modern prokaryotes possessing both FtsZ and ESCRT-III, FtsZ directs cell division, whilst ESCRT-III functions elsewhere, possibly in membrane repair or protein sorting and membrane vesiculation [19]. This suggests that the primordial role of ESCRT-III was membrane stabilisation or repair under cellular stress–a function still conserved in contemporary bacteria [7], archaea [17], and eukaryotes [27,28].

### How ESCRT-III builds conserved protofilaments across evolutionary domains

The canonical ESCRT-III fold comprises 200–250 amino acids with five core helical domains (H1–H5; Figure 2). H1 and H2/H3 form a hairpin-like motif that characterises the superfamily. Three hinges support multidimensional flexing and are crucial for the structural plasticity that defines ESCRT-III polymers. These hinges transition between closed, intermediate, and open conformations enabling ESCRT-III self-assembly into diverse filament types [29,30]. Most ESCRT-IIIs feature an N-terminal motif (H0), possibly helical, that mediates lipid binding. Some also have less conserved C-terminal domains [3] often involved in modulating polymer dynamics, including the recruitment of Vps4 in eukaryotes and archaea. How ESCRT-III subunits typically build protofilaments was revealed through the reconstruction of helical CHMP1B polymers (Figure 2) [31]. Subunits in the open conformation packed side by side with H5 of one subunit binding the hairpin of its neighbour four subunits along (*j*+4). The fundamental nature of this assembly mechanism for ESCRT-III proteins became apparent when bacterial PspA and Vipp1 were shown forming similar protofilaments [3,32,33]. The recent structures of Asgard archaea ESCRT-III protofilaments [24,34] further confirmed this mode of polymerisation as a conserved principle for ESCRT-III proteins across the tree of life with its origins in the LUCA (Figure 2).

### ESCRT-III protofilaments: building blocks that assemble dynamic 2D and 3D polymers

**Figure 3:**
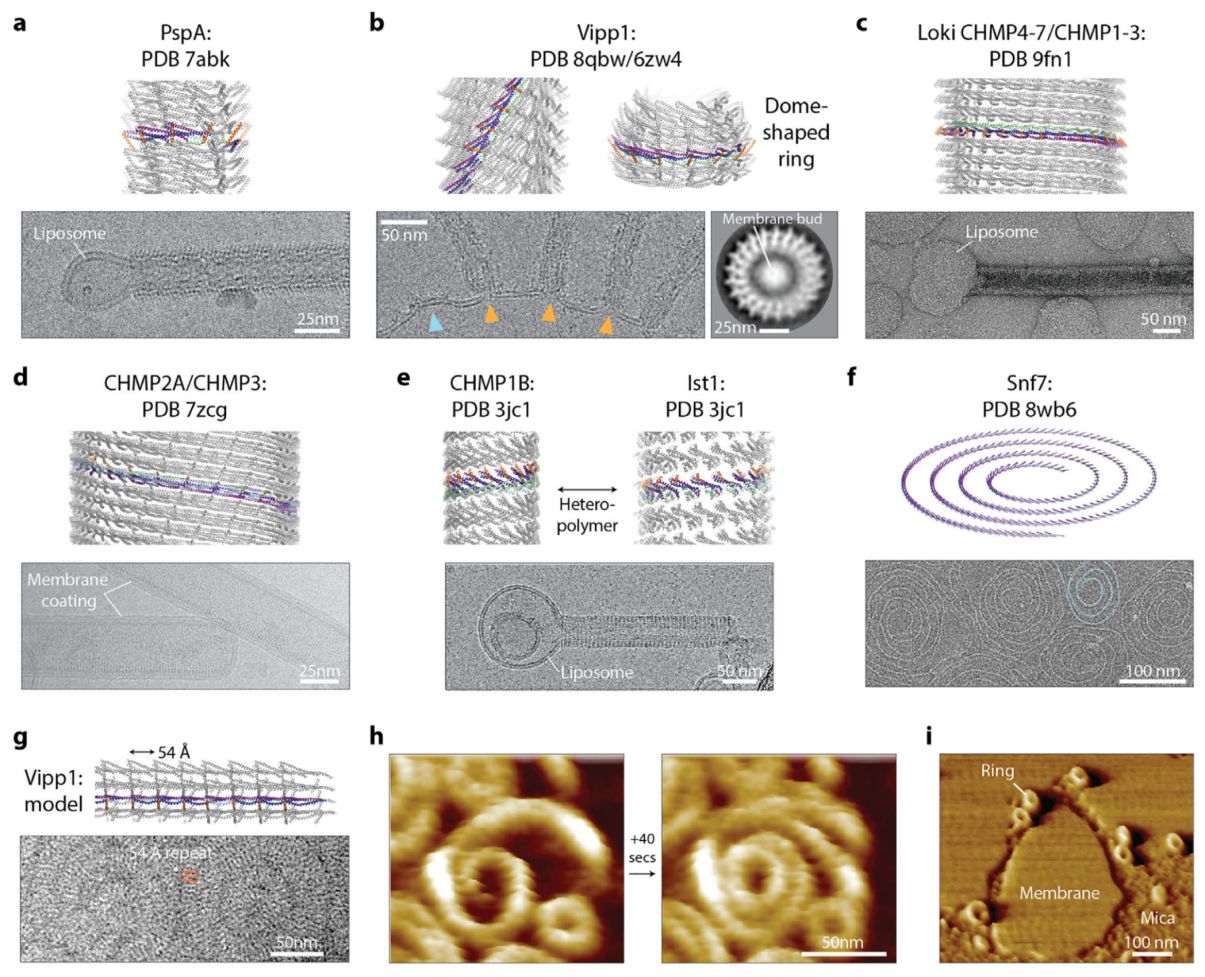
Gallery of ESCRT-III family 2D and 3D polymers. In panels **a**–**e**, structures of selected helical and ring polymers are shown. In each, a single protofilament is coloured like in Figure 2. **(a)** Cryogenic-electron microscopy (cryo-EM) image of PspA tubulating a lipid vesicle [43]. **(b)** (Left) Cryo-EM image showing Vipp1 patches inducing membrane bulging (blue arrow) and helical-like rods tubulating a lipid vesicle (orange arrows) [48]. (Right) Negative-stain electron microscopy (EM) class average of a Vipp1 ring recruited to a lipid monolayer budding membrane within the ring lumen [3]. **(c)** Negative-stain EM image of Loki CHMP4-7 tubulating a 1:1 phosphatidylcholine/phosphatidylserine liposome [34]. **(d)** Cryo-EM image of CHMP2A-CHMP3 forming an inner coating on negatively curved membrane [66] consistent with their established localisation within bud necks. CHMP2A and CHMP3 heterodimerise in the open conformation to form 38- to 49-nm diameter ESCRT-III–like protofilaments angled almost radial to the filament tube axis [66]. **(e)** Cryo-EM image of CHMP1B and IST1 copolymer coating a brominated liposome [77]. CHMP1B is an example of outside membrane binding (at least *in vitro*) whose filaments additionally constrict when Ist1, in a closed conformation, binds their surface forming a helical coating [67]. **(f)** A cryo-EM image showing Snf7 spirals coating a lipid monolayer with a single spiral highlighted in blue. The pseudoatomic model shows how spirals comprise one ESCRT-III protofilament with subunits likely in the open conformation [59]. **(g)** Negative-stain image showing *Nostoc punctiforme* Vipp1 forming 2D sheets and spirals on a lipid monolayer. The structure shows a sheet modelled as four parallel Vipp1 protofilaments associating laterally [49]. **(h)** AFM image time course of Vipp1 forming spiral filaments that usually nucleate at the membrane edge [49]. **(i)** AFM image showing how preformed Vipp1 dome-shaped rings sense and bind to membrane edges where the lipid is perturbed or highly curved [49]. AFM, atomic force microscopy; ESCRT-III, endosomal sorting complex required for transport-III.

ESCRT-III proteins assemble diverse 2D and 3D superstructures including spirals, cones, rings, carpets, and helical filaments and tubes (Figure 3a–i) [30]. ESCRT-III protofilaments are the basic building block for many of these superstructures with the protofilaments often forming heteropolymers or composite filaments with two or more components in eukaryotic systems. Changes in filament composition occur in a sequence, with B-type ESCRT-IIIs typically following A-type ones, resulting in a stepwise increase in polymer curvature and membrane constriction [35–39]. In Asgard archaea, a similar principle has been demonstrated, with B-type ESCRT-III recruiting A-type ones, to yield composite polymers [24]. A similar mechanism appears to have evolved convergently in the Crenarchaeota *Sulfolobus acidocaldarius*, where the Cdv proteins CdvB, CdvB1, and CdvB2 form a composite and stepwise assembled ESCRT-III division ring [40,41]. Bacteria usually have a single PspA copy, so only those lineages (<10%) with PspA gene duplications may have capability for heteropolymer or composite filament formation [5,6]. In contrast, Vipp1 often has paralogues in cyanobacteria [3] making the prospect of composite Vipp1 polymers more feasible, if not likely, given Vipp1 interacts with Vipp2 in the eukaryote *Chlamydomonas reinhardtii* [42].

### Bacterial PspA and Vipp1 polymer types

Whilst the PspA lineage spread across most bacteria, its paralogue Vipp1 emerged in cyanobacteria and subsequently plastid-containing algae and plants (Figure 1a). Both PspA and Vipp1 form variable-diameter helical filaments with and without membrane templates (Figure 3a and b). The ESCRT-III protofilaments they form assemble like CHMP1B except H5 binds the hairpin of its neighbour three subunits along (*j*+3; Figure 2). In addition, H5 simultaneously binds the hairpin of subunits in neighbouring ESCRT-III protofilaments–a property that appears specific to PspA and Vipp1 systems. In *Synechocystis* PspA, helical rods have 18- to 25-nm diameters with ESCRT-III protofilaments angled almost radially to the tube axis. Membrane binding is mediated by an amphipathic N-terminal helix (H0) that lines the inner lumen face [33,43]. Unexpectedly, PspA binds and hydrolyses nucleotide despite lacking classical catalytic motifs [44]. ATP presence promotes PspA plasticity with up to 40-nmdiameter tubes engulfing membrane. Subsequent ATP hydrolysis is linked to the enhanced production of intralumen-like vesicles [43]. However, PspA membrane remodelling *in vitro* is nuanced with membrane fusion activities also observed possibly due to the squeezing and merging of smaller vesicles within PspA tube lumens. In classical PspA systems including *E. coli* [45–47], *B. subtilis* (LiaH) [10], and *M. tuberculosis* (Rc2744c) [13] rings, rod-like ring stacks, cage-like scaffolds, and tubes have been shown to assemble *in vitro* but are yet to be structurally characterised to high resolution. Cellular studies are necessary to understand which polymer types, if not a mix, are physiological, particularly given the diverse range of PspA partners [5,6].

Cyanobacterial Vipp1 helical filaments have ~21- to 29-nm diameters with ESCRT-III protofilaments angled either diagonally or axially, around or along, membrane tubes [48,49]. This shows how Vipp1-derived ESCRT-III protofilaments can twist to coat different membrane planes using the same membrane-binding interface. It also shows how the helical lattice can rotate and flex around different, flat or positive, membrane curvatures. Such capabilities may enable other ESCRT-III proteins to adapt to complex and dynamic 3D membrane landscapes such as the saddle-shaped bud neck of vesicles. The mechanism of membrane binding is mediated by the amphipathic N-terminal helix (H0) stacking in linear arrays around the inner lumen face where it nestles amongst negatively charged lipid head groups. The C-terminal extension (CTE) which distinguishes Vipp1 from PspA locates to the filament outer surface. Although disordered, it promotes helical filament instability by destabilising the conserved hairpin–H5 interface [49]. In this way, the CTE tunes Vipp1 assembly dynamics and promotes fluidity between different polymer forms. In addition, the CTE has been shown to associate with membrane and to affect the membrane fusion capabilities of Vipp1 *in vitro* [50].

Vipp1 also assembles dome-shaped rings with different diameters and symmetries that internalise lipid to create membrane buds (Figure 3b) [3,32]. The rings are formed by the stacking of circularised ESCRT-III protofilaments around the bud axis. Rings of equivalent or sequentially smaller diameter can stack to form cone-like structures with interconnected inner lumens capable of squeezing lipid [3,48]. The rings usefully show i) membrane budding where membrane internalisation is consistent with a capillary action-like mechanism conducted by membrane-binding domains lining the inner lumen [3]. This mechanism is distinct from alternative membrane remodelling strategies such as spiral filament-stored elastic stress minimisation [51]; ii) how ESCRT-III protofilaments can tilt to accommodate different cambers of membrane curvature [52]; iii) how subunits within ESCRT-III protofilaments can flex to assemble rings with variable radii without adjusting subunit number. Similar flex supports changes in diameter of helical PspA rods [43]; iv) how ESCRT-III subunits can have a flexing limit which consequently constrains protofilament curvature [3]. This constraint may explain why multicomponent ESCRT-III systems use a sequence of proteins with different preferred filament curvatures to exert force on membrane over broad curvature ranges [24,35–37]; and v) how ESCRT-III subunits can slide relative to each other to compress or extend the protofilament [43,49,53]. Finally, Vipp1 rings also bind nucleotide [54–56], with hydrolysis modulating *in vitro* ring assembly [32]. However, no link has yet been made between nucleotide turnover and polymerisation dynamics or membrane remodelling capabilities as for PspA [43].

In addition, Vipp1 forms 2D planar sheets or carpets [48,49,57], as well as dynamic spirals on membrane templates reminiscent of Snf7 spirals (Figure 3f and g) [51,58,59]. These Vipp1 spirals can merge laterally to form expansive polygon-shaped membrane coatings [49,60]. Specifically, *Nostoc punctiforme* Vipp1 is recruited to highly curved or perturbed membrane edges where filaments comprising ~4 protofilaments nucleate and curl anticlockwise into spirals that converge centrally (Figure 3h). Here, rings form that protrude and detach, possibly representing nascent dome-shaped ring biogenesis. Importantly, the lattice within the 2D sheets and polygons is geometrically equivalent to an unfurled helical filament or dome-shaped ring with parallel ESCRT-III protofilaments binding laterally [49]. Collectively, this suggests how the same lattice may be used to morph between 2D and 3D forms with only modest interface adjustments required. This phenomenon may be relevant in eukaryotic systems as they transition from planar to budded membrane-bound structures.

### Archaeal ESCRT-III polymers reveal bacterial- and eukaryotic-like features

The discovery of the Cdv system with its role in cell division, vesicle budding, and viral release was an early marker of ESCRT-III in archaea [2,61]. As determined by both reconstituted and cellular systems [62], CdvA and CdvB form a circumferential ring at the division site that serves to recruit CdvB1 and CdvB2 likely as a copolymer. Cell division is triggered upon CdvB degradation by the proteasome [63], with the CdvB1/CdvB2 ring constricting and inducing scission by CdvC-mediated subunit disassembly [41,64] and possibly via supercoiling [65]. High-resolution structures showcasing the architecture and assembly of the Cdv polymer remain outstanding.

In contrast, recent structural studies on ESCRT-III from two Asgard systems [24,34] provide a snapshot into archaeal filament assembly and membrane remodelling capabilities. Both organisms encode ESCRT-IIIA and ESCRT-IIIB that are ancestral to A-type and B-type eukaryotic paralogues, respectively (Figure 2). In Lokiarchaeota, these proteins are called CHMP1-3 and CHMP4-7, and they combine to form 35- to 55-nm helical rods with a spikey surface ultrastructure like PspA (Figure 3c) [34]. However, whilst CHMP1-3 induces morphological changes in the rod including filament constriction, CHMP1-3 was not actually observed in the final cryogenic-electron microscopy (cryo-EM) structure. Instead, only CHMP4-7 forms ESCRT-III protofilaments angled almost radially to the tube with H5 of one subunit binding its neighbour four subunits along, like CHMP1B (Figure 2). The rods were capable of docking with vesicles and internalising them into positively curved membrane tubes (Figure 3c). This finding is reminiscent of membrane invagination within Vipp1 dome-shaped rings by a capillary action–like mechanism [3].

In Heimdallarchaeota archaeon AB_125, ESCRT-IIIB forms filament bundles comprising four twisting protofilaments with H5 of one subunit binding its neighbour four subunits along [24]. However, when bound to low-curvature vesicles, ESCRT-III protofilaments associate laterally to form an arrangement reminiscent of the Loki CHMP4-7/CHMP1-3 helical lattice [34]. Membrane binding is mediated by a loop connecting H3 and H4, and the N-terminus of H1 where a hydrophobic patch corresponding to H0 (Figure 2) may extend into the membrane. This patch is like those in Snf7 (B-type) [59] and CHMP2A-CHMP3 (A-type) [66]. In contrast, ESCRT-IIIA preferentially binds to highly curved membranes where it forms ~31-nm diameter helical filaments angled almost radially to the membrane tube axis, reminiscent of 28-nm diameter A-type CHMP1B polymers [31,67]. Importantly, ESCRT-IIIA/ESCRT-IIIB composite filaments yield an intermediate curvature. This combined with ESCRT-IIIA requiring the presence of ESCRT-IIIB for recruitment to flat membranes, reminiscent of B-type ESCRT-III proteins that are typically recruited early in the membrane remodelling pathway relative to A-type proteins, supports a stepwise membrane remodelling mechanism in Asgard archaea, as proposed for eukaryotic ESCRT-III systems [35–38,40,68].

### Common properties of ESCRT-III membrane remodelling across cellular life

The characterisation of bacterial ESCRT-III systems, combined with recent insights from *in vitro* studies of Asgard systems, highlights some common properties of ESCRT-III. These include i) protofilaments composed of ESCRT-III subunits in an open conformation that interface with neighbouring subunits three or four positions apart (*j*+3 or *j*+4), constitute a common building block for both 2D and 3D polymers (Figure 2); ii) ESCRT-III coats membrane, with the ability to bind either flat or positively curved lipid a prevalent feature across evolutionary lineages, at least under *in vitro* conditions; iii) 3D ESCRT-III polymers, like helical filaments or dome-shaped rings with membrane-binding domains facing the inner cavity, tend to promote membrane internalisation and tubulation that generates positive curvature, at least under *in vitro* conditions (Figure 3a–c and e); iv) both ESCRT-III protofilaments and their assembled higher-order structures can adapt to different membrane orientations and curvatures through relatively minor lattice modifications, achieved by subunit flexing or intersubunit sliding or torsion; and v) ESCRT-III subunits and the higher-order structures they build have flexing limits that pave the way for stepwise pathways that exploit the preferred curvatures of individual ESCRT-III protofilaments.

### ESCRT-III membrane remodelling in bacterial cells

**Figure 4:**
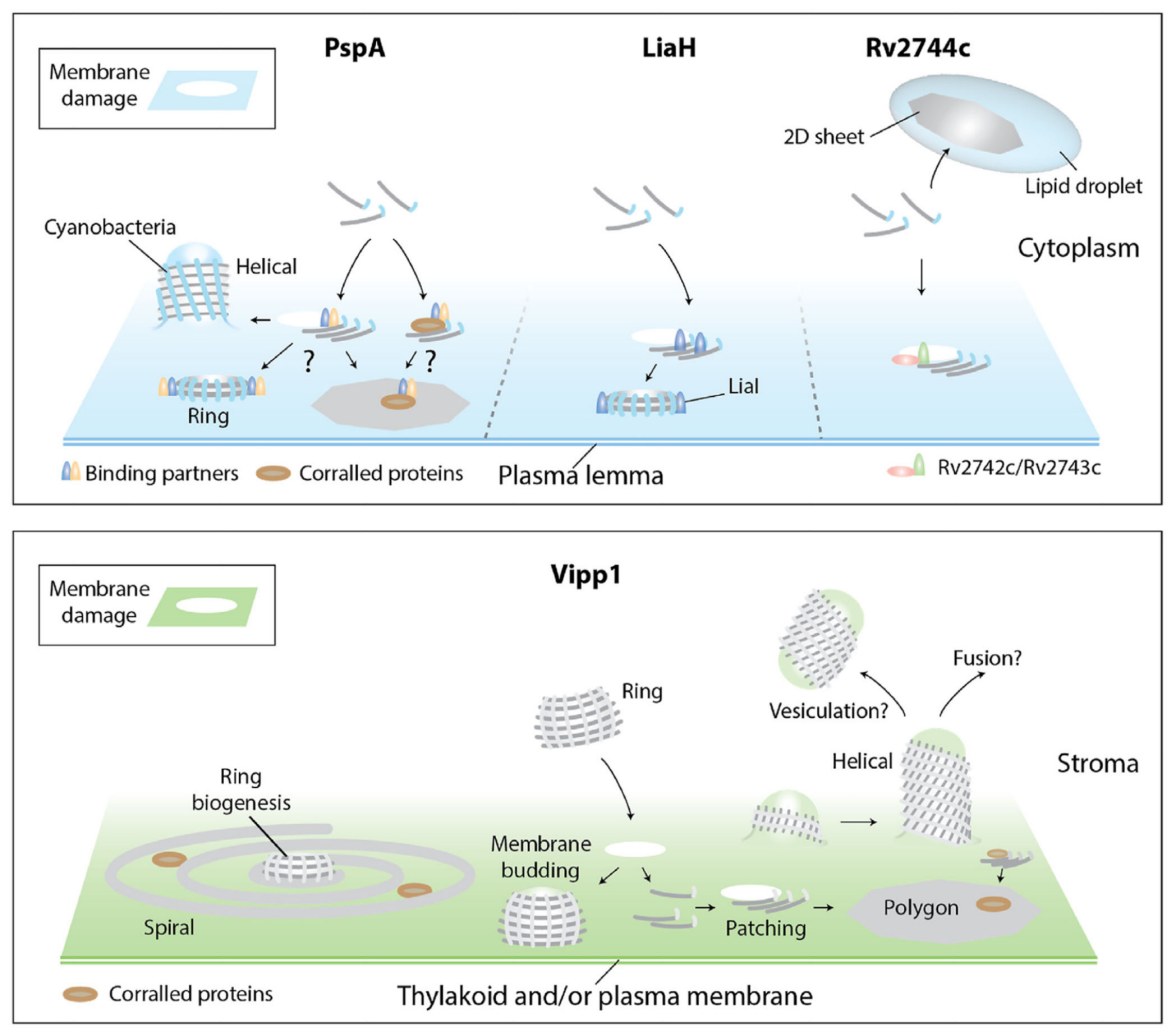
PspA and Vipp1 membrane remodelling and repair in bacterial cells. Processes shown are not exhaustive, are derived from current knowledge and are speculative as the types of PspA and Vipp1 polymers active in cells are ultimately unknown. In Proteobacteria such as *Escherichia coli*, PspA forms part of the *psp* operon comprising *pspABCDEFG*. Membrane stress detection by inner membrane proteins PspB and PspC triggers PspA release from PspF, an AAA + ATPase in the cytosol, thereby promoting PspA recruitment to the membrane [7]. In *Bacillus subtilis*, inner membrane protein LiaI dynamically scans the membrane for stress. Upon detection, it recruits LiaH to the membrane forming static foci that presumably mitigate stress-induced damage [12]. It is not known whether PspA, LiaH, Rv2744c, or other homologues form 2D polymers like Vipp1 polygons and carpets. Depending on the system, Vipp1 may be localised to the thylakoid, plasma membrane, or both. It may promote the fusion of opposing membranes or possibly support bridges between the plasma membrane and the thylakoid [32].

In bacteria, the types of PspA-like polymers forming in the cell are unknown and may differ across large evolutionary distances, such as between *E. coli, B. subtilis, M. tuberculosis*, and cyanobacteria with their extensive photosynthetic membrane networks. PspA and LiaH are activated by membrane stressors such as antibiotics, hyperosmotic shock, solvents, detergents, the Tat pathway [9], membrane-inserted proteins like secretin channels [69], or the cup-like oligomers assembled by Stomatin, Prohibitin, Flotillin, and HflK/C (SPFH)-domain proteins [11,70]. Speculatively, PspA, and LiaH may form rings [10,46] or cage-like scaffolds [45] that may stack into rods [47], all capable of sealing or budding membrane, like Vipp1 (Figure 4). In cyanobacteria, helical structures [33] may assemble over perturbed membrane regions, budding and squeezing the membrane. A reasonable hypothesis is that PspA forms 2D carpet-like structures or spirals like Vipp1, particularly as the carpets reduce proton permeability in liposomes [57], and PspA mitigates loss of proton motive force in diverse bacteria [71]. The ability of PspA to form high-density zones linked with membrane-remodelling events hints at similar membrane-coating capabilities [33]. In *M. tuberculosis*, the PspA homologue Rv2744c complexes with the membrane protein Rv2743c which in turn interacts with Rv2742. Together they may contribute to envelope integrity maintenance in response to surface stress [14]. Rv2744c also localises to lipid droplets, regulating their number and size [13], and may form a carpet-like surface coating similar to how Vipp1 coats lipid monolayers [3]. This function may have distant evolutionary convergence with CHMP1B and Ist1 which facilitate the tethering of lipid droplets to peroxisomes [72].

The Vipp1 cellular membrane stress response may be actioned by 2D spirals or sheets [49,60] corralling around membrane complexes like Photosystem II [73] and forming a protective seal. Additionally, Vipp1 dome-shaped rings or helical-like structures may form around, or be recruited to, perturbed membrane (Figure 3i) [49], where they may induce membrane budding and repair by lipid leaflet squeezing [3]. Alternatively, such recruited Vipp1 structures may disassemble, morphing into protective 2D sheets [57]. Vipp1 buds transition to form free-coated vesicles and tubes *in vitro* [48] with similar structures glimpsed in *Synechocystis* and *Chlamydomonas* (when overexpressed) [32], respectively. Coated vesicles may serve as a lipid transport mechanism by harnessing Vipp1 fusogenic properties to dock with opposing membrane surfaces [48,50,74–76].

## Conclusion

It is now established that bacterial PspA and Vipp1 proteins are homologous to ESCRT-III, unifying this superfamily across the tree of life. Archaeal ESCRT-III is also understood to be evolutionarily related to eukaryotic forms, with Asgard ESCRT-III systems likely playing a formative role in the origin of eukaryotic cells and the emergence of complex endomembrane systems. Recently, this expanding prokaryotic ESCRT-III field has provided exciting snapshots of ESCRT-III structure and mechanism, revealing conserved principles. Furthermore, structural analyses (Figure 3) indicate a tendency for prokaryotic ESCRT-III to bind and promote flat or positively curved membrane, similar to eukaryotic CHMP1B *in vitro* [31,67,77]. This observation creates an apparent contradiction with certain eukaryotic ESCRT-III proteins, including CHMP1B itself, which often localises to sites of reverse topology (negatively curved) membrane in the cell, such as within budding necks. This finding supports the hypothesis that eukaryotic ESCRT-III proteins may be i) restricted to the mouth of bud necks rather than forming long-coiled polymers operating within the neck lumen where curvature is most negative [78]; and ii) recruited to membrane geometries that deviate from their preferred membrane-binding topology, so that minimising filament frustration becomes an important factor in generating mechanical forces for membrane scission [79,80]. Here, the capillary action–like mechanism used by prokaryotic ESCRT-III rings and helical polymers to draw in membrane could supply additional membrane constriction force [3,34]. Such an effect would be mediated by ring or helical filaments containing just a few turns since extensively coiled polymers are not expected to form at bud sites [78]. Ultimately, key questions remain including the role of ESCRT-III in Asgard archaea and the types of PspA and Vipp1 polymer present in cells. With ESCRT-II–related components identified in cyanobacteria [81], it is possible that other parts of the ESCRT pathway await discovery in bacteria. Finally, the binding of both archaeal and eukaryotic systems to DNA and chromatin may hint at yet another important role for ESCRT-III [34,82].

## Data Availability

No data was used for the research described in the article.

## References

[1] Hurley JH (2015). ESCRTs are everywhere. EMBO J.

[2] Lindås A-C, Karlsson EA, Lindgren MT, Ettema TJG, Bernander R (2008). A unique cell division machinery in the Archaea. Proc Natl Acad Sci.

[3] Liu J, Tassinari M, Souza DP, Naskar S, Noel JK, Bohuszewicz O, Buck M, Williams TA, Baum B, Low HH (2021). Bacterial Vipp1 and PspA are members of the ancient ESCRT-III membrane-remodeling superfamily. Cell.

[4] Moody ERR, Álvarez-Carretero S, Mahendrarajah TA, Clark JW, Betts HC, Dombrowski N, Szánthó LL, Boyle RA, Daines S, Chen X (2024). The nature of the last universal common ancestor and its impact on the early Earth system. Nat Ecol Evol.

[5] Ravi J, Anantharaman V, Chen SZ, Brenner EP, Datta P, Aravind L, Gennaro ML (2024). The phage shock protein (PSP) envelope stress response: discovery of novel partners and evolutionary history. mSystems.

[6] Popp PF, Gumerov VM, Andrianova EP, Bewersdorf L, Mascher T, Zhulin IB, Wolf D (2022). Phyletic distribution and diversification of the phage shock protein stress response system in bacteria and archaea. mSystems.

[7] Joly N, Engl C, Jovanovic G, Huvet M, Toni T, Sheng X, Stumpf MPH, Buck M (2010). Managing membrane stress: the phage shock protein (Psp) response, from molecular mechanisms to physiology. FEMS Microbiol Rev.

[8] Krüger L, Gaskell-Mew L, Graham S, Shirran S, Hertel R, White MF (2024). Reversible conjugation of a CBASS nucleotide cyclase regulates bacterial immune response to phage infection. Nat Microbiol.

[9] Bernal-Cabas M, Miethke M, Antelo-Varela M, Aguilar Suárez R, Neef J, Schön L, Gabarrini G, Otto A, Becher D, Wolf D (2020). Functional association of the stress-responsive LiaH protein and the minimal TatAyCy protein translocase in Bacillus subtilis. Biochim Biophys Acta Mol Cell Res.

[10] Wolf D, Kalamorz F, Wecke T, Juszczak A, Mäder U, Homuth G, Jordan S, Kirstein J, Hoppert M, Voigt B (2010). In-depth profiling of the LiaR response of *Bacillus subtilis*. J Bacteriol.

[11] Scholz AS, Baur SSM, Wolf D, Bramkamp M (2021). An Stomatin, Prohibitin, Flotillin, and HflK/C-domain protein required to link the phageshock protein to the membrane in Bacillus subtilis. Front Microbiol.

[12] Domínguez-Escobar J, Wolf D, Fritz G, Höfler C, Wedlich-Söldner R, Mascher T (2014). Subcellular localization, interactions and dynamics of the phage-shock protein-like Lia response in Bacillus subtilis. Mol Microbiol.

[13] Armstrong RM, Adams KL, Zilisch JE, Bretl DJ, Sato H, Anderson DM, Zahrt TC (2016). Rv2744c is a PspA ortholog that regulates lipid droplet homeostasis and nonreplicating persistence in Mycobacterium tuberculosis. J Bacteriol.

[14] Datta P, Ravi J, Guerrini V, Chauhan R, Neiditch MB, Shell SS, Fortune SM, Hancioglu B, Igoshin OA, Gennaro ML (2015). The Psp system of Mycobacterium tuberculosis integrates envelope stress-sensing and envelope-preserving functions. Mol Microbiol.

[15] Zhang L, Sakamoto W (2013). Possible function of VIPP1 in thylakoids. Plant Signal Behav.

[16] Rütgers M, Schroda M (2013). A role of VIPP1 as a dynamic structure within thylakoid centers as sites of photosystem biogenesis?. Plant Signal Behav.

[17] Bidle KA, Kirkland PA, Nannen JL, Maupin-Furlow JA (2008). Proteomic analysis of Haloferax volcanii reveals salinity-mediated regulation of the stress response protein PspA. Microbiology (N Y).

[18] Lu Z, Fu T, Li T, Liu Y, Zhang S, Li J, Dai J, Koonin EV, Li G, Chu H (2020). Coevolution of eukaryote-like Vps4 and ESCRT-III subunits in the asgard archaea. mBio.

[19] Makarova KS, Tobiasson V, Wolf YI, Lu Z, Liu Y, Zhang S, Krupovic M, Li M, Koonin EV (2024). Diversity, origin, and evolution of the ESCRT systems. mBio.

[20] Liu J, Cvirkaite-Krupovic V, Commere P-H, Yang Y, Zhou F, Forterre P, Shen Y, Krupovic M (2021). Archaeal extracellular vesicles are produced in an ESCRT-dependent manner and promote gene transfer and nutrient cycling in extreme environments. ISME J.

[21] Liu J, Cvirkaite-Krupovic V, Baquero DP, Yang Y, Zhang Q, Shen Y, Krupovic M (2021). Virus-induced cell gigantism and asymmetric cell division in archaea. Proc Natl Acad Sci.

[22] Hatano T, Palani S, Papatziamou D, Salzer R, Souza DP, Tamarit D, Makwana M, Potter A, Haig A, Xu W (2022). Asgard archaea shed light on the evolutionary origins of the eukaryotic ubiquitin-ESCRT machinery. Nat Commun.

[23] Spang A, Saw JH, Jørgensen SL, Zaremba-Niedzwiedzka K, Martijn J, Lind AE, van Eijk R, Schleper C, Guy L, Ettema TJG (2015). Complex archaea that bridge the gap between prokaryotes and eukaryotes. Nature.

[24] Souza DP, Espadas J, Chaaban S, Moody ERR, Hatano T, Balasubramanian M, Williams TA, Roux A, Baum B (2025). Asgard archaea reveal the conserved principles of ESCRT-III membrane remodeling. Sci Adv.

[25] Rodrigues-Oliveira T, Wollweber F, Ponce-Toledo RI, Xu J, Rittmann SK-MR, Klingl A, Pilhofer M, Schleper C (2023). Actin cytoskeleton and complex cell architecture in an Asgard archaeon. Nature.

[26] Santana-Molina C, del Saz-Navarro Dm, Devos DP (2023). Early origin and evolution of the FtsZ/tubulin protein family. Front Microbiol.

[27] Dai E, Meng L, Kang R, Wang X, Tang D (2020). ESCRT-III–dependent membrane repair blocks ferroptosis. Biochem Biophys Res Commun.

[28] Stempels FC, Jiang M, Warner HM, Moser M-L, Janssens MH, Maassen S, Nelen IH, de Boer R, Jiemy WF, Knight D (2023). Giant worm-shaped ESCRT scaffolds surround actin-independent integrin clusters. JCB (J Cell Biol).

[29] Schlösser L, Sachse C, Low HH, Schneider D (2023). Conserved structures of ESCRT-III superfamily members across domains of life. Trends Biochem Sci.

[30] McCullough J, Frost A, Sundquist WI (2018). Structures, functions, and dynamics of ESCRT-III/Vps4 membrane remodeling and fission complexes. Annu Rev Cell Dev Biol.

[31] McCullough J, Clippinger AK, Talledge N, Skowyra ML, Saunders MG, Naismith TV, Colf LA, Afonine P, Arthur C, Sundquist WI (2015). Structure and membrane remodeling activity of ESCRT-III helical polymers. Science.

[32] Gupta TK, Klumpe S, Gries K, Heinz S, Wietrzynski W, Ohnishi N, Niemeyer J, Spaniol B, Schaffer M, Rast A (2021). Structural basis for VIPP1 oligomerization and maintenance of thylakoid membrane integrity. Cell.

[33] Junglas B, Huber ST, Heidler T, Schlösser L, Mann D, Hennig R, Clarke M, Hellmann N, Schneider D, Sachse C (2021). PspA adopts an ESCRT-III-like fold and remodels bacterial membranes. Cell.

[34] Melnikov N, Junglas B, Halbi G, Nachmias D, Zerbib E, Gueta N, Upcher A, Zalk R, Sachse C, Bernheim-Groswasser A (2025). The Asgard archaeal ESCRT-III system forms helical filaments and remodels eukaryotic-like membranes. EMBO J.

[35] Jiang X, Harker-Kirschneck L, Vanhille-Campos C, Pfitzner A-K, Lominadze E, Roux A, Baum B, Šarić A (2022). Modelling membrane reshaping by staged polymerization of ESCRT-III filaments. PLoS Comput Biol.

[36] Pfitzner A-K, Mercier V, Jiang X, Moser von Filseck J, Baum B, Šarić A, Roux A (2020). An ESCRT-III polymerization sequence drives membrane deformation and fission. Cell.

[37] Meadowcroft B, Palaia I, Pfitzner A-K, Roux A, Baum B, Šarić A (2022). Mechanochemical rules for shape-shifting filaments that remodel membranes. Phys Rev Lett.

[38] Saksena S, Wahlman J, Teis D, Johnson AE, Emr SD (2009). Functional reconstitution of ESCRT-III assembly and disassembly. Cell.

[39] Teis D, Saksena S, Emr SD (2008). Ordered assembly of the ESCRT-III complex on endosomes is required to sequester cargo during MVB formation. Dev Cell.

[40] Hurtig F, Burgers TCQ, Cezanne A, Jiang X, Mol FN, Traparić J, Pulschen AA, Nierhaus T, Tarrason-Risa G, Harker-Kirschneck L (2023). The patterned assembly and stepwise Vps4-mediated disassembly of composite ESCRT-III polymers drives archaeal cell division. Sci Adv.

[41] De Franceschi N, Blanch-Jover A, Dekker C (2025). Interaction hierarchy among Cdv proteins drives recruitment to membrane necks. eLife.

[42] Theis J, Niemeyer J, Schmollinger S, Ries F, Rütgers M, Gupta TK, Sommer F, Muranaka LS, Venn B, Schulz-Raffelt M (2020). VIPP2 interacts with VIPP1 and HSP22E/F at chloroplast membranes and modulates a retrograde signal for *HSP22E/F* gene expression. Plant Cell Environ.

[43] Junglas B, Hudina E, Schönnenbeck P, Ritter I, Heddier A, Santiago-Schübel B, Huesgen PF, Schneider D, Sachse C (2024). Structural plasticity of bacterial ESCRT-III protein PspA in higher-order assemblies. Nat Struct Mol Biol.

[44] Ohnishi N, Zhang L, Sakamoto W (2018). VIPP1 involved in chloroplast membrane integrity has GTPase activity in vitro. Plant Physiol.

[45] Standar K, Mehner D, Osadnik H, Berthelmann F, Hause G, Lünsdorf H, Brüser T (2008). PspA can form large scaffolds in *Escherichia coli*. FEBS Lett.

[46] Hankamer BD, Elderkin SL, Buck M, Nield J (2004). Organization of the AAA+ adaptor protein PspA is an oligomeric ring. J Biol Chem.

[47] Male AL, Oyston PCF, Tavassoli A (2014). Self-assembly of Escherichia coli phage shock protein A. Adv Microbiol.

[48] Junglas B, Kartte D, Kutzner M, Hellmann N, Ritter I, Schneider D, Sachse C (2024). Structural basis for Vipp1 membrane binding: from loose coats and carpets to ring and rod assemblies. Nat Struct Mol Biol.

[49] Naskar S, Merino A, Espadas J, Singh J, Roux A, Colom A, Low HH (2024). Mechanism for Vipp1 spiral formation, ring biogenesis, and membrane repair. Nat Struct Mol Biol.

[50] Hennig R, West A, Debus M, Saur M, Markl J, Sachs JN, Schneider D (2017). The IM30/Vipp1 C-terminus associates with the lipid bilayer and modulates membrane fusion. Biochim Biophys Acta Bioenerg.

[51] Chiaruttini N, Redondo-Morata L, Colom A, Humbert F, Lenz M, Scheuring S, Roux A (2015). Relaxation of loaded ESCRT-III spiral springs drives membrane deformation. Cell.

[52] Harker-Kirschneck L, Baum B, Šarić A (2019). Changes in ESCRT-III filament geometry drive membrane remodelling and fission in silico. BMC Biol.

[53] Banjade S, Tang S, Shah YH, Emr SD (2019). Electrostatic lateral interactions drive ESCRT-III heteropolymer assembly. eLife.

[54] Siebenaller C, Schlösser L, Junglas B, Schmidt-Dengler M, Jacob D, Hellmann N, Sachse C, Helm M, Schneider D (2021). Binding and/or hydrolysis of purine-based nucleotides is not required for IM30 ring formation. FEBS Lett.

[55] Junglas B, Siebenaller C, Schlösser L, Hellmann N, Schneider D (2020). GTP hydrolysis by Synechocystis IM30 does not decisively affect its membrane remodeling activity. Sci Rep.

[56] Ohnishi N, Sugimoto M, Kondo H, Shioya K, Zhang L, Sakamoto W (2022). Distinctive in vitro ATP hydrolysis activity of AtVIPP1, a chloroplastic ESCRT-III superfamily protein in arabidopsis. Front Plant Sci.

[57] Junglas B, Orru R, Axt A, Siebenaller C, Steinchen W, Heidrich J, Hellmich UA, Hellmann N, Wolf E, Weber SAL (2020). IM30 IDPs form a membrane-protective carpet upon super-complex disassembly. Commun Biol.

[58] Jukic N, Perrino AP, Humbert F, Roux A, Scheuring S (2022). Snf7 spirals sense and alter membrane curvature. Nat Commun.

[59] Liu M, Liu Y, Song T, Yang L, Qi L, Zhang Y-Z, Wang Y, Shen Q-T (2024). Three-dimensional architecture of ESCRT-III flat spirals on the membrane. Proc Natl Acad Sci U S A.

[60] Pan S, Gries K, Engel BD, Schroda M, Haselwandter CA, Scheuring S (2024). The cyanobacterial protein VIPP1 forms ESCRT-III-like structures on lipid bilayers. Nat Struct Mol Biol.

[61] Samson RY, Obita T, Freund SM, Williams RL, Bell SD (2008). A role for the ESCRT system in cell division in archaea. Science (1979).

[62] Pulschen AA, Mutavchiev DR, Culley S, Sebastian KN, Roubinet J, Roubinet M, Risa GT, van Wolferen M, Roubinet C, Schmidt U (2020). Live imaging of a hyperthermophilic archaeon reveals distinct roles for two ESCRT-III homologs in ensuring a robust and symmetric division. Curr Biol.

[63] Tarrason Risa G, Hurtig F, Bray S, Hafner AE, Harker-Kirschneck L, Faull P, Davis C, Papatziamou D, Mutavchiev DR, Fan C (1979). The proteasome controls ESCRT-III–mediated cell division in an archaeon. Science.

[64] Blanch Jover A, De Franceschi N, Fenel D, Weissenhorn W, Dekker C (2022). The archaeal division protein CdvB1 assembles into polymers that are depolymerized by CdvC. FEBS Lett.

[65] Harker-Kirschneck L, Hafner AE, Yao T, Vanhille-Campos C, Jiang X, Pulschen A, Hurtig F, Hryniuk D, Culley S, Henriques R (2022). Physical mechanisms of ESCRT-III–driven cell division. Proc Natl Acad Sci.

[66] Azad K, Guilligay D, Boscheron C, Maity S, De Franceschi N, Sulbaran G, Effantin G, Wang H, Kleman J-P, Bassereau P (2023). Structural basis of CHMP2A–CHMP3 ESCRT-III polymer assembly and membrane cleavage. Nat Struct Mol Biol.

[67] Nguyen HC, Talledge N, McCullough J, Sharma A, Moss FR, Iwasa JH, Vershinin MD, Sundquist WI, Frost A (2020). Membrane constriction and thinning by sequential ESCRT-III polymerization. Nat Struct Mol Biol.

[68] Pfitzner A-K, Zivkovic H, Bernat-Silvestre C, West M, Peltier T, Humbert F, Odorizzi G, Roux A (2023). Vps60 initiates alternative ESCRT-III filaments. JCB (J Cell Biol).

[69] Naskar S, Hohl M, Tassinari M, Low HH (2021). The structure and mechanism of the bacterial type II secretion system. Mol Microbiol.

[70] Ma C, Wang C, Luo D, Yan L, Yang W, Li N, Gao N (2022). Structural insights into the membrane microdomain organization by SPFH family proteins. Cell Res.

[71] Wang M, Chan EWC, Wan Y, Wong MH, Chen S (2021). Active maintenance of proton motive force mediates starvation-induced bacterial antibiotic tolerance in Escherichia coli. Commun Biol.

[72] Chang C-L, Weigel AV, Ioannou MS, Pasolli HA, Xu CS, Peale DR, Shtengel G, Freeman M, Hess HF, Blackstone C (2019). Spastin tethers lipid droplets to peroxisomes and directs fatty acid trafficking through ESCRT-III. JCB (J Cell Biol).

[73] Bryan SJ, Burroughs NJ, Shevela D, Yu J, Rupprecht E, Liu L, Mastroianni G, Xue Q, Llorente-Garcia I, Leake MC (2014). Localisation and interactions of the Vipp1 protein in cyanobacteria. Mol Microbiol.

[74] Thurotte A, Schneider D (2019). The fusion activity of IM30 rings involves controlled unmasking of the fusogenic core. Front Plant Sci.

[75] Heidrich J, Thurotte A, Schneider D (2017). Specific interaction of IM30/Vipp1 with cyanobacterial and chloroplast membranes results in membrane remodeling and eventually in membrane fusion. Biochim Biophys Acta Biomembr.

[76] Hennig R, Heidrich J, Saur M, Schmüser L, Roeters SJ, Hellmann N, Woutersen S, Bonn M, Weidner T, Markl J (2015). IM30 triggers membrane fusion in cyanobacteria and chloroplasts. Nat Commun.

[77] Moss FR, Lincoff J, Tucker M, Mohammed A, Grabe M, Frost A (2023). Brominated lipid probes expose structural asymmetries in constricted membranes. Nat Struct Mol Biol.

[78] Adell MAY, Migliano SM, Upadhyayula S, Bykov YS, Sprenger S, Pakdel M, Vogel GF, Jih G, Skillern W, Behrouzi R (2017). Recruitment dynamics of ESCRT-III and Vps4 to endosomes and implications for reverse membrane budding. eLife.

[79] Bertin A, de Franceschi N, de la Mora E, Maity S, Alqabandi M, Miguet N, di Cicco A, Roos WH, Mangenot S, Weissenhorn W (2020). Human ESCRT-III polymers assemble on positively curved membranes and induce helical membrane tube formation. Nat Commun.

[80] Moser von Filseck J, Barberi L, Talledge N, Johnson IE, Frost A, Lenz M, Roux A (2020). Anisotropic ESCRT-III architecture governs helical membrane tube formation. Nat Commun.

[81] Yilmazer I, Vetrano P, Eicke S, Abt MR, Traverso E, Morosinotto T, Zeeman SC, Ramundo S, Sharma M (2023). A conserved ESCRT-II-like protein participates in the biogenesis and maintenance of thylakoid membranes. bioRxiv.

[82] Nachmias D, Melnikov N, Zorea A, Sharon M, Yemini R, Depicchoto Y, Tsirkas I, Aharoni A, Frohn B, Schwille P (2023). Asgard ESCRT-III and VPS4 reveal conserved chromatin binding properties of the ESCRT machinery. ISME J.

[83] Frohn BP, Härtel T, Cox J, Schwille P (2022). Tracing back variations in archaeal ESCRT-based cell division to protein domain architectures. PLoS One.

